# Strengthening healthcare providers’ leadership capabilities, interprofessional collaboration, and systems thinking: a conceptualization of the Clinical Scholars program impact

**DOI:** 10.1186/s12909-024-06240-1

**Published:** 2024-11-07

**Authors:** Tara Carr, Scott Rosas, Cheryl Noble, Michelle Song, Claudia S. P. Fernandez, Kathleen Brandert, Kathy Donnald, Giselle Corbie, Gaurav Dave

**Affiliations:** 1 Abacus Evaluation, University of North Carolina, Chapel Hill, NC USA; 2 Center for Health Equity Research, Department of Social Medicine, University of North Carolina, Chapel Hill, NC USA; 3 Concept Systems Incorporated (CSI), Ithaca, NY USA; 4 Department of Maternal and Child Health, University of North Carolina, Chapel Hill, NC USA; 5 Department of Health Promotion, University of Nebraska Medical Center, Omaha, NE USA; 6 Department of Medicine, University of North Carolina, Chapel Hill, NC USA

**Keywords:** Clinical scholars, Concept mapping, Equity, Healthcare, Interprofessional collaboration, Leadership, Systems

## Abstract

**Background:**

Healthcare provider leadership programs represent an intervention opportunity to support advancement of the national system of care and an evaluation of their program impacts is needed. Between 2016 and 2023, the Robert Wood Johnson Foundation (RWJF) funded Clinical Scholars (CS), a three-year equity-centered leadership training program for US healthcare providers. CS recruited participants (referred to as Fellows) in cohorts and engaged them as members of interprofessional teams to transform their careers and the health of their communities. The aim of this study was to evaluate Fellows’ perspectives on the success of CS, specifically the program elements and their importance for community well-being and sustainability.

**Methods:**

We used the mixed methods group concept mapping (GCM) approach to evaluate Fellows’ perspectives on program success. First, we conducted the qualitative phases of brainstorming, sorting, and rating with Fellows. Secondly, we conducted the quantitative phases using multi-dimensional scaling and hierarchical cluster analysis and integrated the sorting and rating information from each Fellow to develop a series of concept maps. Finally, we conducted the interpretation phase to synthesize findings. Fellows (*N* = 177) across five cohorts were invited to participate in the study.

**Results:**

Fifty-seven Fellows (32%) completed one or more GCM phases. A conceptual map emerged, consisting of seven thematic clusters, which showed that program value could be attributed to the following elements: *“Resources”*,* “Wicked Problem Impact Project (WPIP) Support”*,* “Curriculum”*,* “Thinking Bigger”*,* “Leadership Training”*, *“Networking”*, and *“Teamwork.”* The pattern match showed that all seven clusters were highly rated by Fellows across the *Community Well-being Impact* and *Sustainability* domains.

**Conclusion:**

Study findings support the value of the RWJF-CS program strategy of long-term investment in the development of healthcare leaders with applied skills in interprofessional collaboration who will be prepared to continue addressing complex, multi-faceted challenges in the system of care.

**Supplementary Information:**

The online version contains supplementary material available at 10.1186/s12909-024-06240-1.

## Background

Despite a substantial financial expenditure, the United States (US) lags other high-income nations with respect to multiple healthcare quality indicators [1]. The World Bank, World Health Organization (WHO) and Organization for Economic Co-operation and Development (OECD) have indicated that a foundational component for the provision of a high-quality national healthcare system is a skilled and competent health workforce that is supported and motivated [2]. Training programs that contribute to developing the healthcare workforce represent an intervention opportunity to advance the system of care as part of a comprehensive strategy nationally and globally [3, 4].

Training the healthcare workforce should incorporate continuing education as an option to advance and expand healthcare delivery. Continuing education programs can focus on critical skills beyond clinical knowledge such as providers’ ability to work in cross-disciplinary teams, develop a personal leadership style, and attain other professional capabilities that foment an integrated vision of care. Interpersonal collaboration, the extent to which different healthcare professionals work well together, has the potential to improve the quality of health care that providers deliver [5] and increase the likelihood of desired health outcomes. Clinical leadership can also improve healthcare quality and innovation [2, 6], and serve as a vehicle to transform healthcare and community services [7, 8]. Centering health equity in the system of care is pivotal to addressing adverse social determinants of health (SDOH) [9–12]. Few leadership programs target these skills in an integrated, multi-year, team-based, and applied learning format [13–19]. Moreover, impact evaluations of healthcare leadership programs are often absent.

From 2016 to 2023, the Robert Wood Johnson Foundation (RWJF) funded the Clinical Scholars National Leadership Institute, also known as Clinical Scholars (CS), a three-year equity-centered leadership training program for US healthcare providers [13, 20, 21]. CS delivered professional training and development through an intensive in-person program and a robust distance-based “Continuous Learning Program” [13, 22]. Fellows were recruited in cohorts and engaged in the program as members of interdisciplinary teams. Teams collaborated across sectors to tackle significant, complex health problems they identified in their communities, referred to as a “Wicked Problem”, and subsequently developed and implemented science- and community-based solutions [23]. The CS leadership team published a detailed description of the program elements and their experiences in developing the team and program [22].

As part of a comprehensive program evaluation [24], the primary aim of this present study was to evaluate Fellows’ perspectives on the success of the CS program. We used group concept mapping (GCM), a mixed methods research approach, to gather, aggregate, confirm and integrate the specific knowledge and perspectives of Fellows as to the program elements that made CS successful. We also sought to explore the importance of those identified elements with respect to (1) advancing health equity in the communities in which Fellows work, and (2) sustaining their work after completion of CS.

## Methods

### Study design

We selected GCM because of its participatory nature allowing for numerous ideas to be elicited and synthesized across all CS cohorts. GCM is a mixed methods research approach that integrates familiar qualitative group processes (brainstorming, synthesizing and categorizing ideas, and assigning value ratings) with multivariate statistical analyses to help a group describe its ideas on any topic of interest and represent these ideas visually through a series of related maps. The shared conceptual model can then be used as a tool for evaluation. The GCM process typically requires the participants to complete three main data collection activities: (1) brainstorming a set of ideas relevant to the topic of interest; (2) individually sorting these ideas into themes/groups based on perceived similarity; and (3) rating each idea on one or more dimensions. The GCM process is driven by relevant partners, ranging from initial brainstorming, to the eventual identification and naming of clusters of thought, and then to the analysis and interpretation of quantitative maps.

**Fig. 1:**
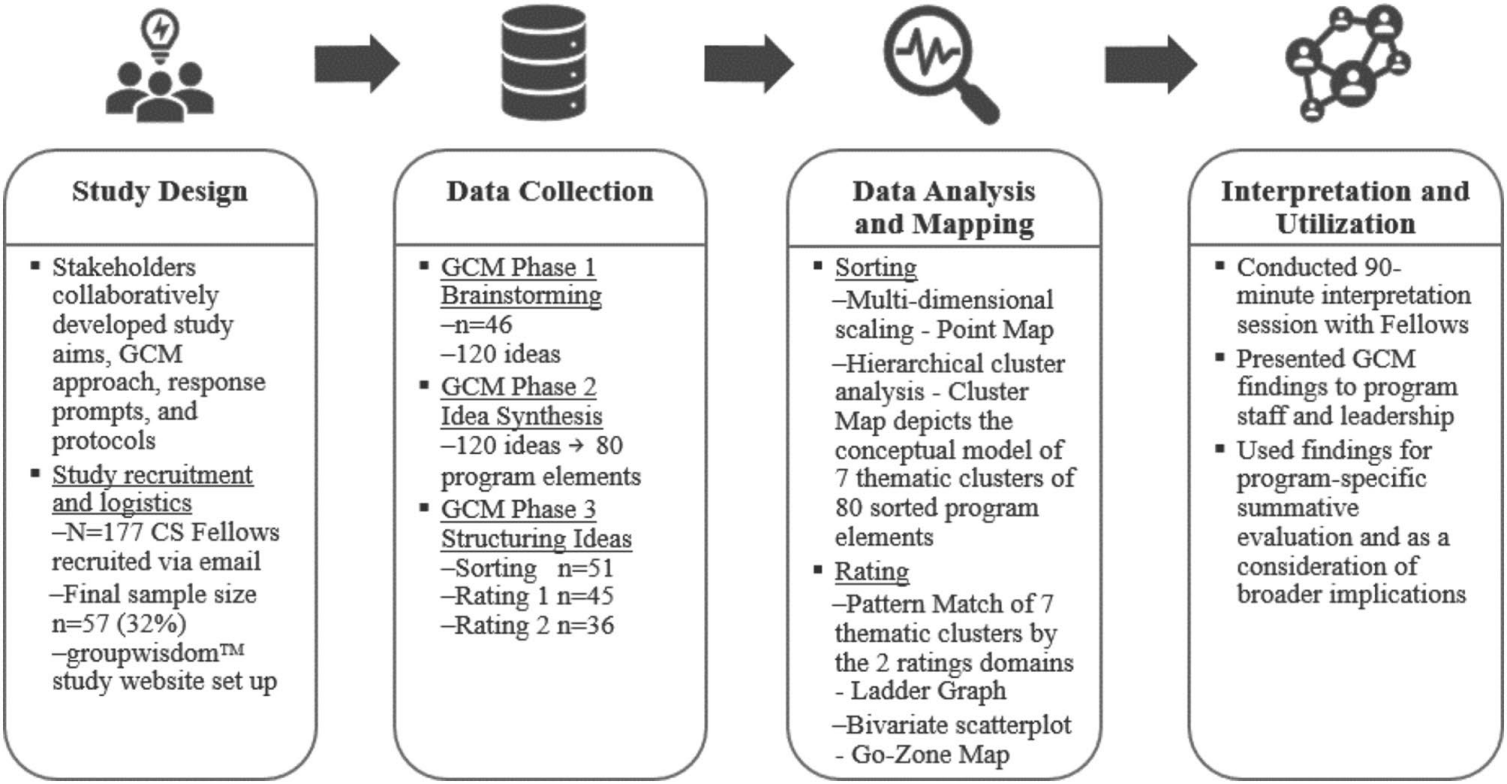
Study overview. The study was developed to evaluate Fellows’ perspectives on the success of the CS program. The four steps of the study, which used a GCM methodology, were design, data collection, data analysis and mapping, and interpretation and utilization. Within the data collection step, the number of Fellows in each GCM phase is displayed

The evaluation team (herein referred to as the study team) collaborated with CS program staff and leadership to develop the overall study approach and protocols during spring 2022. The study overview with the number of Fellows in each step of data collection is displayed in Fig. 1. While collaboration was integral to ensure that the study team had adequate context to conduct the GCM study, steps were taken to ensure impartiality. The study team independently conducted the study in spring-summer 2022 and neither the program staff nor leadership were involved in data collection or analysis. External experts at Concept Systems Incorporated (CSI) provided technical consulting assistance on GCM, supported the dedicated study-specific groupwisdom™ website where data collection tasks were completed online [25], independently ran analyses to ensure the integrity of the study teams’ findings, and facilitated the interpretation session. Fellows as well as members of the program staff and leadership helped the study team with the interpretation of resultant findings, where the latter additionally provided important information for dissemination including considerations and broader implications for advanced healthcare provider training, research, and policy.

### Sample size

We invited via email all Fellows (*N* = 177) who engaged in any cohort program activities to participate in the study. The final sample size was 57 (32%).

### Data collection

#### Demographic characteristics

We collected the following demographic information from participating Fellows: the year in which they started CS (e.g., cohort), race, ethnicity, clinical discipline, and primary employment sector.

#### GCM phase 1. brainstorming

Fellows were asked to respond to the focus prompt: “From your perspective, an element that made Clinical Scholars successful was…”. They were asked to provide as many ideas to the focus prompt as they would like and were able to view the ideas submitted by others. Forty-six Fellows provided 120 responses.

#### GCM phase 2. Idea synthesis

Following the steps outlined by Kane and Trochim, the study team used the following criteria in reviewing the preliminary responses to produce a final list of program elements: (a) relevance to or within the scope of the focus prompt; (b) redundancy or duplication; (c) clarity of meaning; and (d) relative appropriateness for the sorting and rating tasks to be completed [26]. After applying the review criteria to the brainstorming responses, the study team finalized a list of 80 program elements.

#### GCM phase 3. Structuring ideas

In phase three, the study team recontacted Fellows and asked them to participate in two tasks over a month to structure the information: sorting and rating. Fellows were able to save their work and return to the study website multiple times to complete the activities.

#### Sorting

Fellows were asked to sort all 80 program elements from the brainstorming task into themes/groups based upon similarity in meaning to one another in a way that made sense to them. Fellows were also asked to name each of the themes/groups based on the content of the elements they contained. Fifty-one Fellows completed sorting.

#### Rating

Fellows then were asked to rate the importance of each of the 80 program elements on two dimensions: *Community Well-being Impact* (Rating 1) and *Sustainability* (Rating 2). Fellows received the following instructions: “Please rate the importance of each item in regard to making an *impact on community well-being”* and “Please rate the importance of each item in regard to continuing to advance health equity in your community *after completing Clinical Scholars.”* They were instructed to provide their rating using a four point, Likert-type scale (1 = Not important at all; 4 = Essential). Forty-five Fellows completed the *Community Well-being Impact* ratings and 36 completed the *Sustainability* ratings.

### Data analysis

Descriptive analysis was used to assess the sample characteristics. We then performed the GCM analyses with the Concept System^®^ groupwisdom™ platform [25] which uses multi-dimensional scaling (MDS) and hierarchical cluster analysis to compile, analyze, and aggregate the sorting data, and develop a series of easily readable point and cluster concept maps [25]. Once the appropriate cluster arrangement was determined for the sorting data, the ratings data on the *Community Well-being Impact* and *Sustainability* domains were integrated to further explore the values assigned by the Fellows to each of the program elements through pattern matching and bivariate analysis. First, the average *Community Well-being Impact* and *Sustainability* rating was computed for each program element. Next, each item-level average was averaged again based on the item’s cluster membership. Comparisons between the ratings were made at both the cluster and program element levels for both the *Community Well-being Impact* and *Sustainability* domains. Lastly, we created a Go-Zones map to compare the *Community Well-being Impact* and *Sustainability* ratings among all the rated program elements.

## Results

### Sample characteristics

The sample characteristics are presented in Table 1. Fellows (*n* = 57) were drawn from all five cohorts with the largest representation from the last two program cohorts where approximately one-third were from 2020 (cohort 5; 36.8%) and 2019 (cohort 4; 31.6%) each, and the remainder from the other three, 2016–2018 (cohorts 1–3; 31.5%). There was a diversity of clinical disciplines with the majority being physicians (28.0%), nurse/nurse practitioners (19.3%), and social workers (17.5%). The most represented primary employment sectors were university/college (35.0%) and hospital (19.3%).

**Table 1:** Sample characteristics (*n* = 57 fellows)

Sample Characteristics	*n*	%
**Clinical Scholars cohort year**		
2016 (cohort 1)	8	14.0
2017 (cohort 2)	8	14.0
2018 (cohort 3)	2	3.5
2019 (cohort 4)	18	31.5
2020 (cohort 5)	21	37.0
Total	57	100.0
**Race**		
Asian	8	14.3
Bi-Racial or Multi-Racial	3	5.4
Black/African American	4	7.0
Native Hawaiian or Pacific Islander	3	5.4
White/Caucasian	37	66.1
Other	1	1.8
Total	56	100.0
**Ethnicity**		
Hispanic/Latino	5	9.1
Not Hispanic/Latino	50	90.9
Total	55	100.0
**Clinical discipline**		
Certified Nurse Midwife	1	1.8
Dentist	1	1.8
Dietician/Nutritionist	1	1.8
Nurse/Nurse Practitioner	11	19.3
Occupational Therapist	2	3.5
Pharmacist	3	5.2
Physician	16	28.0
Physician Assistant	1	1.8
Psychologist	7	12.3
Social Worker	10	17.5
Veterinarian/Vet Nurse	4	7.0
Total	57	100.0
**Primary employment sector**		
Community Based Organization	4	7.0
Federally Qualified Health Center	5	8.8
Government	2	3.5
Hospital	11	19.3
Nonprofit	4	7.0
Outpatient Care	5	8.8
Self-Employed	2	3.5
Tribal	2	3.5
University/College	20	35.0
Veterinary Services	1	1.8
Other	1	1.8
Total	57	100.0

### Sorting results and concept maps

Our analyses of the sort data generated point and cluster concept maps which show the perceived relationships of the 80 program elements synthesized from brainstorming (GCM Phase 1). In these maps and the ratings map (described in the ‘Rating results’ section below), the program elements are depicted with points and labeled with numbers which can be matched with a table for further exploration [see Additional file 1].

#### Point map

**Fig. 2:**
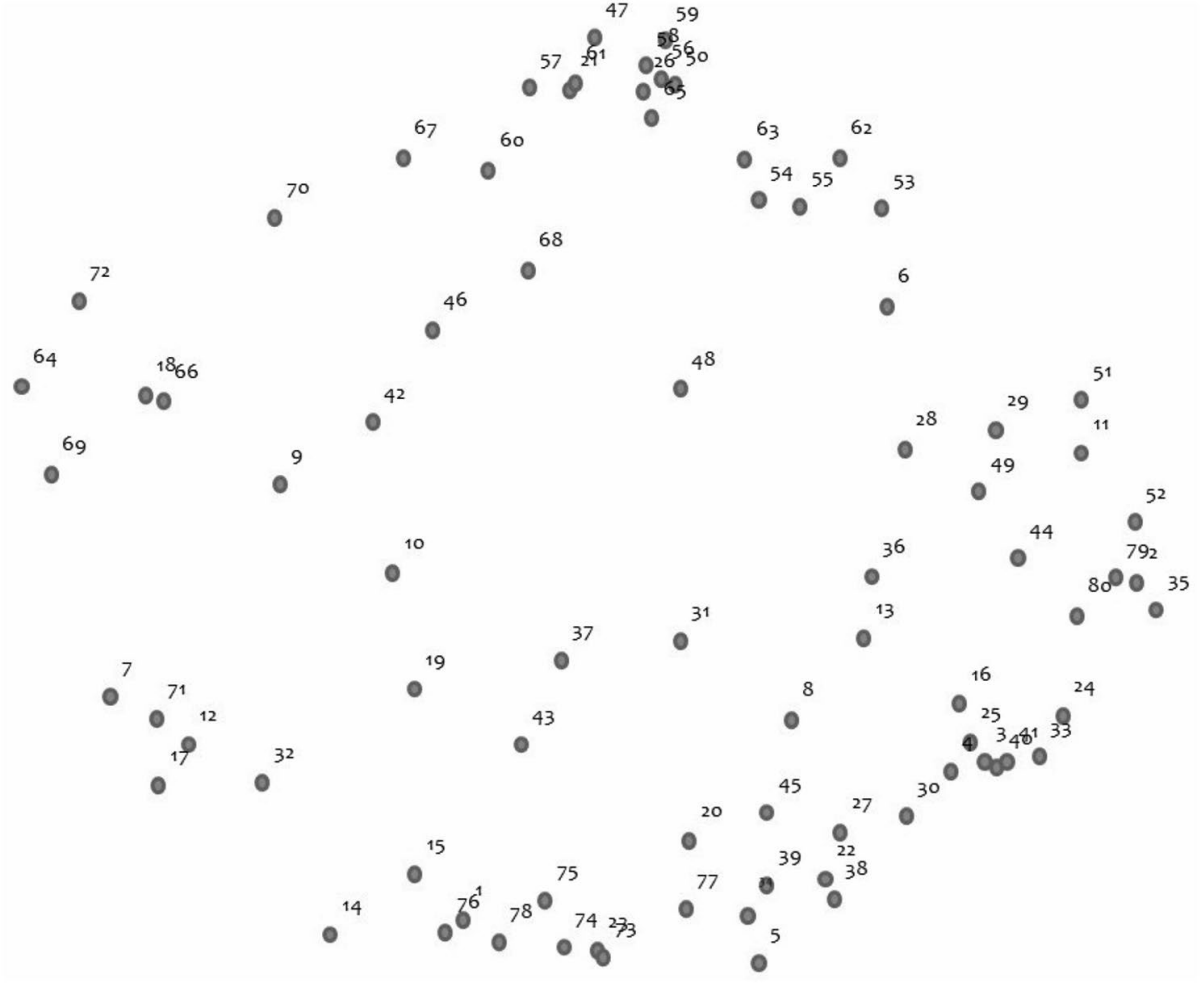
Point map of the 80 program elements. Each numbered point represents one of the 80 program elements (Fellow responses) from brainstorming (GCM Phase 1). Program elements that were more frequently sorted together (GCM Phase 3) into themes/groups and are closer in meaning appear closer together in the map

The MDS analysis generated a point map which shows each of the 80 program elements in a two-dimensional array as a point which reflects a meaningful arrangement of the content as perceived by the Fellows (see Fig. 2). Program elements that are closer together were sorted more often by Fellows into the same theme/group, indicating that they are closer in meaning to one another. Conversely, program elements that are farther apart were sorted less frequently by the Fellows and are less close in meaning. Based on the similarity matrix from sort data, the MDS analysis of the similarity matrix converged after 13 iterations with a final stress value of 0.2570. This stress value, which is within the generally accepted range of 0.205 to 0.365 [26–29], provided evidence of a good fit between the raw sort data and the point map representation [28, 30].

#### Cluster map

**Fig. 3:**
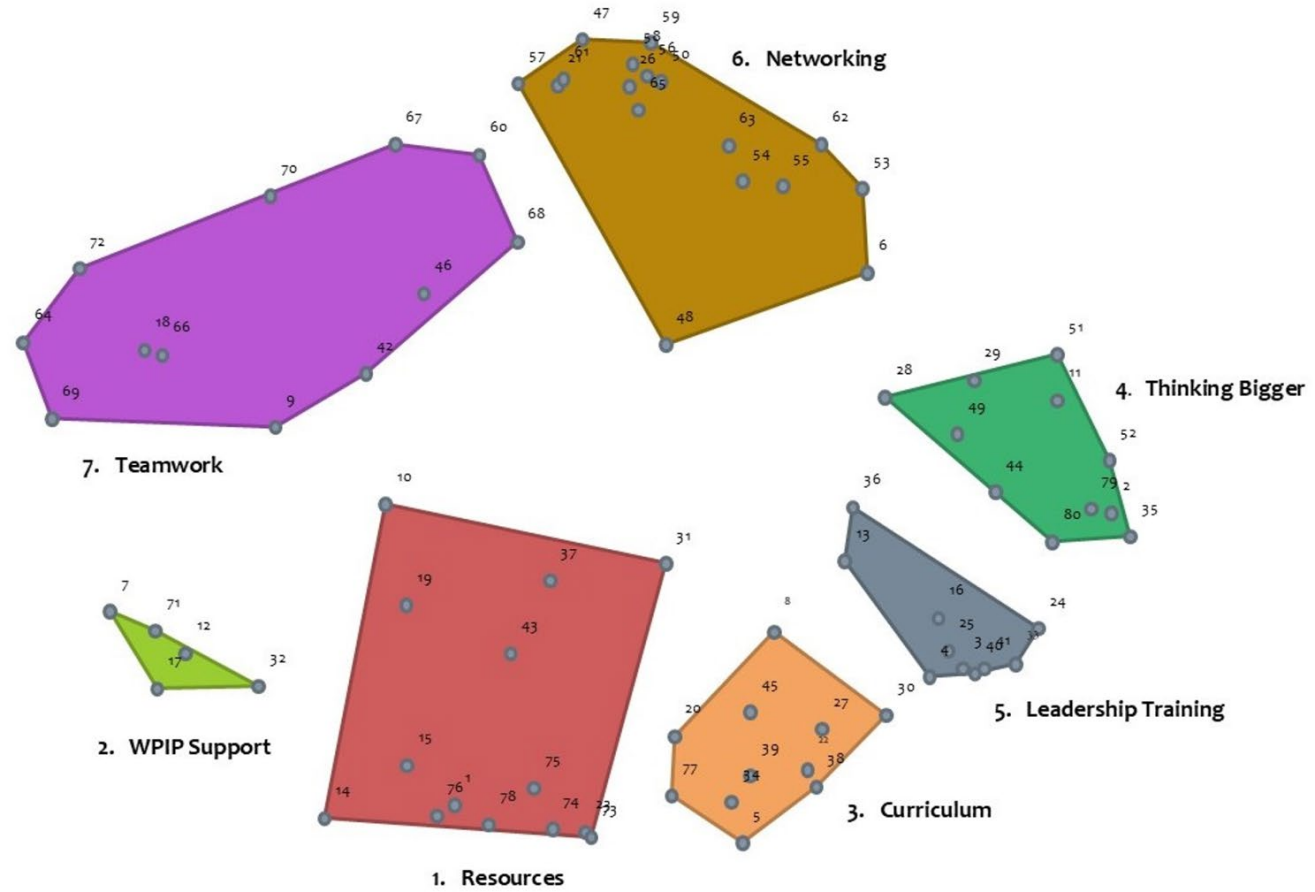
Cluster map of the Clinical Scholars program success areas (clusters). The cluster map shows how the 80 program elements, depicted by numbered points, are related via higher level concepts in a spatially-oriented way. A seven-cluster solution emerged as the optimal solution based on the sorting data. These seven areas of program success can be considered as a meaningful framework when discussing the value of Clinical Scholars from the Fellows’ perspective

The hierarchical cluster analysis produced a cluster map which illustrates how the program elements, depicted by the same numbered points as in the point map, are related via higher level concepts in a spatially-oriented way (see Fig. 3). The cluster map shows a seven-cluster arrangement that emerged as the optimal solution based on the sorting data and suggests that these areas of success can be considered as a meaningful framework when discussing the value of CS from the Fellows’ perspective. The cluster names indicate the theme expressed in the program elements within each of the respective clusters: *1.“Resources”*, *2.“Wicked Problem Impact Project (WPIP) Support”*, *3.“Curriculum”*, *4.“Thinking Bigger”*, *5.“Leadership Training”*, *6.“Networking”*, and *7.“Teamwork*.*”* Table 2 lists the seven clusters in no particular order with their descriptions.

**Table 2:** Description of the Clinical Scholars’ success areas (clusters)

Cluster name	Cluster description
1. Resources	focused on the human, technical, administrative, and expertise elements of CS and the recognition of the value of these supports to the Fellows’ success
2. WPIP Support	emphasized the support provided to the Fellows in carrying out the Wicked Problem Impact Project (WPIP)
3. Curriculum	described the materials, tools, technologies, and experiences that collectively constituted the learning plan for Fellows
4. Thinking Bigger	emphasized the ways in which the program impacted and affected the Fellows’ growth and development
5. Leadership Training	described the leadership development processes and outcomes of CS
6. Networking	emphasized the inter-and intra-personal elements of CS and its impact on the Fellows
7. Teamwork	reflected the process of working together as a team within the context of the program and WPIPs

### Rating results

#### Pattern match

**Fig. 4:**
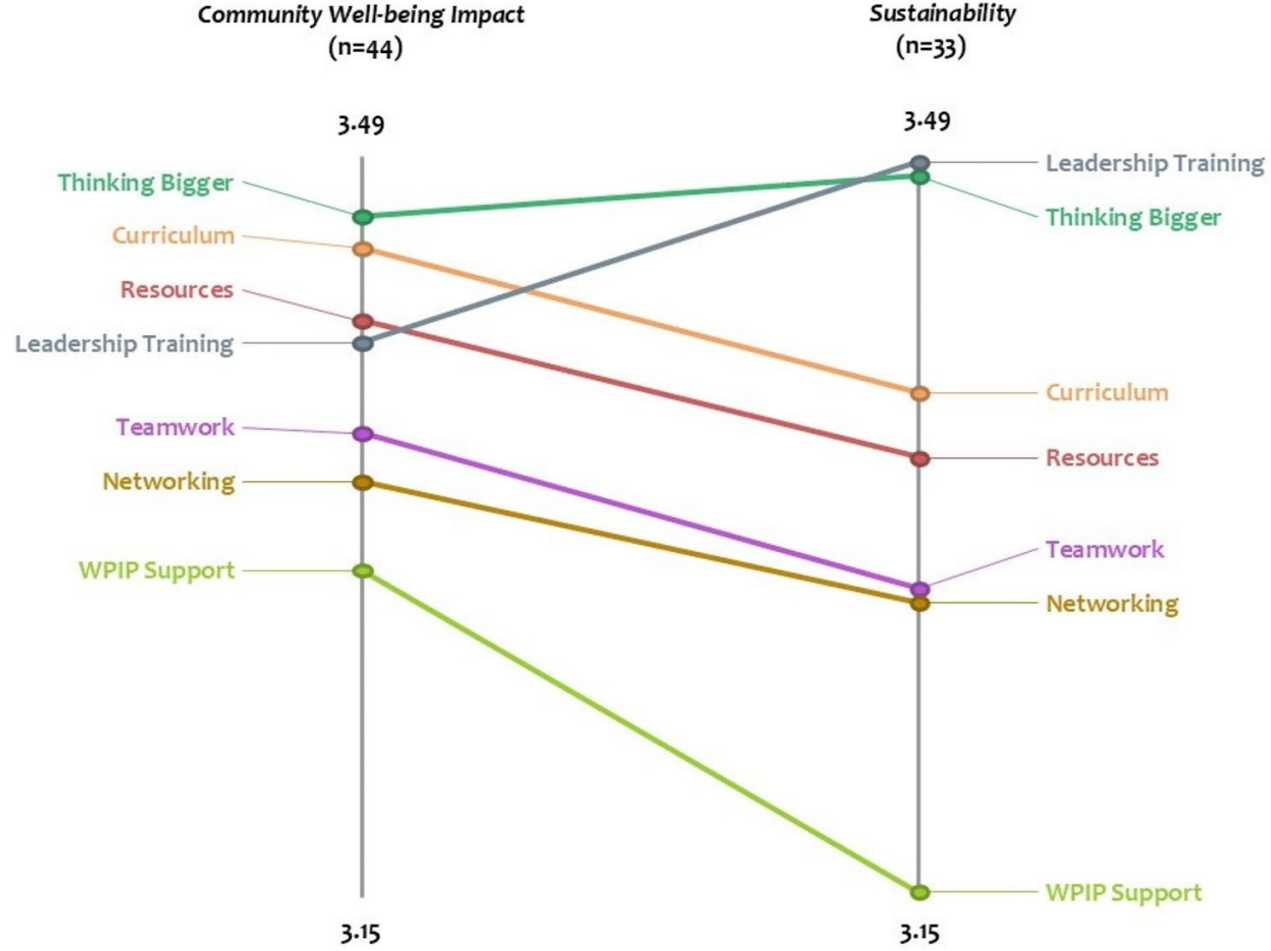
Pattern Match graph comparing average cluster ratings between *Community Wellbeing Impact* and *Sustainability* domains. On average, participants ranked the seven areas of program success (clusters) as important to *Community Well-being Impact* and *Sustainability* and the order of the rankings for both domains were very similar. One difference we observed was that “*Thinking Bigger*”, a systems-perspective, ranked the highest for *Community Well-being Impact* but second highest for *Sustainability* behind “*Leadership Training*”

Using the rating results, we generated pattern matches to compare the average cluster ratings between the *Community Well-being Impact* and *Sustainability* domains. In Fig. 4, the pattern match graph (also referred to as a ladder graph) shows that the average cluster ratings’ order was similar between the two domains. The main pattern difference we observed was that for the *Community Well-being Impact* domain “*Thinking Bigger*” had the highest average cluster rating but had the second highest ranking for *Sustainability* behind “*Leadership Training*.” The graph also shows the correlation between the average domain ratings for each cluster. In this case, the overall correlation was 0.86, which indicates that Fellows’ perceptions of *Community Well-being Impact* were strongly aligned with their perceptions of *Sustainability*. The degree of slope of the lines connecting concepts on the left (*Community Well-being Impact*) to the same concept on the right (*Sustainability*) illustrates the degree of alignment.

#### Go-Zone map

**Fig. 5:**
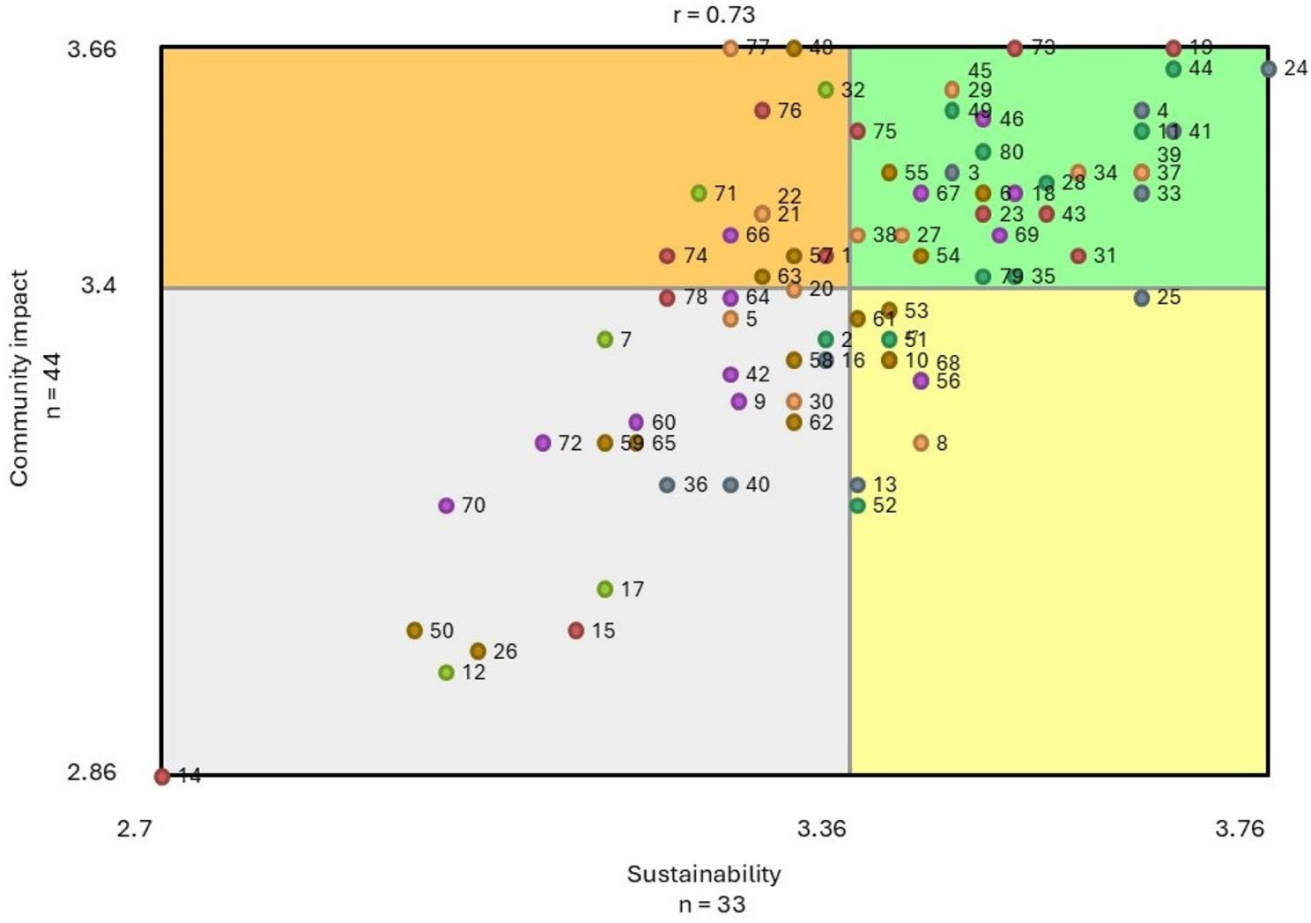
Go-Zone map representing the comparison of *Community-Wellbeing Impact* and *Sustainability* ratings for program elements (separate from the clusters), colour-coded by cluster. There were 32 program elements (40%) in the green Go-Zone suggesting that Fellows viewed them as important to making an impact on well-being in their respective communities and sustaining health equity. The correlation between the two domains (*r* = 0.73) indicates a moderately strong linear relationship – program elements that rated higher on average in importance to making an impact on community well-being were also rated higher on average in importance to sustaining equity

We used a Go-Zone map, a bi-variate scatterplot, to compare the *Community Well-being Impact* and *Sustainability* ratings among the program elements (separate from the clusters). The program elements are presented in quadrants based on a comparison of community well-being impact and sustainability as perceived by the Fellows, with the lines separating the bivariate scatterplot into quadrants determined by the mean values of the two ratings (see Fig. 5). The four quadrants are: (1) higher importance for community well-being impact/higher importance for sustainability (“Go- Zone”, green); (2) higher importance for community well-being impact/lower importance for sustainability (orange); (3) lower importance for community well-being impact/higher importance for sustainability (yellow); and (4) lower importance for community well-being impact/lower importance for sustainability (grey).

Encouragingly, there were 32 statements (40%) in the Go-Zone suggesting these were program elements Fellows viewed as important to both making an impact on well-being in their respective communities and sustaining health equity. These program elements were distributed across six of the seven clusters (areas of program success) represented in the cluster map (see Fig. 3). The correlation between the average ratings of *Community Well-being Impact* and *Sustainability* for each program element was *r* = 0.73, suggesting a moderately strong linear relationship such that those program elements which were rated higher on average in terms of importance to making an impact on well-being in Fellows’ respective communities were also rated higher on average in importance to sustaining equity.

## Discussion

Our key resultant study finding was the seven-cluster solution of the 80 program elements that Fellows brainstormed, which can be considered as a meaningful framework of the CS program success from their perspective: *“Resources”*,* “Wicked Problem Impact Project (WPIP) support”*, *“Curriculum”*, *“Thinking Bigger”*, *“Leadership Training”*, *“Networking”*, and *“Teamwork*.*”* We also sought to understand how Fellows viewed the importance of the identified program elements with respect to both community well-being impact and sustaining their work after the CS program. The pattern match showed that all seven clusters were highly rated by Fellows across and a high correlation of their ratings of the clusters’ importance between the *Community Well-being Impact* and *Sustainability* domains, with the main difference being that *“Thinking Bigger”* was perceived as the primary important element for the former and *“Leadership Training”* for the latter.

Research evidence continues to highlight a global need for interventions designed to equip healthcare providers with practical leadership training to address complex health system problems and exercise interprofessional collaboration. A review conducted by Mianda and Voce [31] investigating clinical leadership interventions for frontline healthcare providers identified 24 papers, all of which described interventions that arose in high-income countries. The review showed that interventions in included papers most often aimed to develop clinical leadership skills and competence in quality improvement primarily among medical doctors and nurses, through various educational techniques including self-directed, enquiry based- or problem-based learning, in-service training, coaching, and mentoring, but did not emphasize interprofessional collaboration, lasted between a few days to several months, and did not explicitly indicate a health-equity focus [31]. In a cross-sectional study conducted by Phillips et al. [32] that surveyed nurses’ knowledge, confidence, and behaviors relative to integrating SDOH into nursing practice across a large regional healthcare system in the US, fewer than 43% reported being knowledgeable on any of the surveyed SDOHs and with respect to perceived barriers and facilitators, respondents stressed the need for interdisciplinary education, stronger collaborative partnerships, and more information on the role of social workers [32]. Overall, our study findings support the value of a RWJF-CS program strategy of long-term investment in the development of healthcare leaders skilled in interprofessional collaboration and poised to address complex, multi-faceted, challenges in the system of care.

### Limitations and strengths

This study does have limitations. The sample size was small and study findings may not fully reflect the perspectives and experiences of all program Fellows. The benefit of the participatory nature of GCM may be dampened if a representative cross-section of a population is not captured or response rate is low [33]. The universe of ideas to create a conceptual map may be limited and perceptions of experiences may be skewed. Our response rate was approximately one-third of all CS Fellows, which is not largely divergent from rates reported in other GCM studies conducted with health and education professionals and policy experts [34–38]. Additionally, our sample represented a wide cross-section of program cohorts, clinical disciplines, and employment sectors. In this sense, the areas of program success (clusters) that emerged from the study appear to be perceived as important across diverse healthcare disciplines and employment sectors. Moreover, given that we were evaluating program elements, the universe of ideas might be more constrained than would be the case for GCM with other topics. Likely, the program elements brainstormed, and sorting would have been the same. Where our response rate may have affected our findings differently is with the ranking process. Potentially, there were differences in experiences and perceptions of the program between those who responded and those who did not and as such, those who did not respond may have ranked the importance of program elements lower and/or differently. In-person data collection during on-site program activities might have increased our study participation and would be a recommended strategy for future evaluation, but this approach was not possible given the geographic dispersion of Fellows and limited opportunity to convene them all after program completion. To mitigate such limitations and unknowns in the evaluation of leadership development programs, communicating the importance of long-term participation and involving Fellows in the conceptualization could have made the study aims and process more salient, and countered fatigue from other evaluation and program asks, thereby increasing participation. Another limitation is that program implementation factors, such as the COVID-19 pandemic, which occurred in the middle of the CS program and resulted in a pivot to a mostly online program delivery mode, may also have affected participant perception of usefulness of different program elements. Finally, the generalizability of findings is a limitation given that this study was conducted as part of a summative evaluation for a US-based program.

Despite these limitations, our study has multiple strengths. We collected study data online which allowed for expanded Fellow participation across cohorts in different GCM phases. As such, the study sample characteristics reflect participation from all five cohorts, 11 healthcare professions, and 11 employment sectors. Our study employed GCM, a structured mixed methods research approach, which allowed for group conceptualization among participants, CS Fellows themselves, of the elements that made the program successful. Furthermore, we conducted a 90-minute online facilitated interpretation session where participants were invited to provide their feedback on and review analytical findings [39, 40]. We found that a key program component was *“Thinking Bigger*,*”* which indicates Fellows place a high value on developing and applying a systems-perspective to individual or team-based initiatives. Further study could investigate why and how this perspective prepares providers to navigate and drive change in their day-to-day work, and if this effect can be evidenced in the health system itself or the health outcomes in the community. Future research on CS community well-being impacts and sustainability, or similar continuing education and health leadership development programs, is needed to incorporate and understand the perspectives of others in the health system or the community beyond the perspective of the implementing teams. This understanding would provide a valuable dimension of how the program projects and solutions implemented to address ongoing challenges of “Wicked Problems” are viewed by the communities most impacted.

### Implications

While almost twenty years have passed since Trochim and Kane described concept mapping’s relevance to health care [41], it remains an underutilized method in health research [42]. Our study’s GCM research approach provides steps that can be adapted by other training initiatives designed for healthcare professionals to evaluate the impact of their interventions. Further, there is an increasing recognition of the importance of developing leaders equipped to address complex systems challenges through collaborative action [43]. Recently, the National Academies of Science, Engineering, and Medicine (US) convened a workshop on health systems science education which in part explored how training can prepare learners to work together within health systems, and improve patient care, population health, and health professional well-being [44]. Overall, our study findings contribute practical program strategies for health care systems decision-makers, including national academies and institutes of health, healthcare organizations, accreditation bodies, and funders, to further support recommendations, policies, programs, and research which align with and can build on the CS approach – the fusion of equity-centered and systems thinking leadership training directed at interdisciplinary and collaborative healthcare provider teams to address complex health and wellbeing issues.

By extension, our study findings may also be relevant to other disciplines such as education [45–47] and urban planning [48] whose work has broad SDOH implications. As with healthcare, both education and urban planning are interdisciplinary in nature, requiring collaboration between professionals focused on research, policy, advocacy, and practice to successfully address SDOH. For example, the integration of the program elements identified in this study into leadership training initiatives within the education field would allow leaders to more effectively tackle complex systems challenges, like the school-to-prison pipeline [49–53], collaboratively and interdisciplinarily. Additionally, a CS-leadership training program approach with cohorts of urban planners, policy makers, public health practitioners, and health care providers would better position them to collaboratively conceptualize and address the complex inter-connections and dynamics between the built environment, urban services, and population health outcomes [48].

## Conclusion

A comprehensive strategy of multiple intervention approaches, carried out at all socioecological levels, and aimed at improving healthcare processes and outcomes is needed to advance the nation’s system of care. Our study findings indicate that the Clinical Scholars program, which takes an equity-centered training approach to increase healthcare professionals’ leadership skills and interprofessional collaboration through a long-term, practice- and team-based format, is a mechanism to increase providers’ capacity to addresses large, complex systemic issues in the healthcare sector. Future research is needed to understand how the program can be effectively adapted and implemented to expand program reach and sustainability.

## Electronic supplementary material

Below is the link to the electronic supplementary material.

Supplementary Material 1

## Data Availability

The datasets generated and/or analysed during the current study are not publicly available due to being part of a program evaluation but are available from the corresponding author on reasonable request.

## Abbreviations

CHER: Center for Health Equity Research
CSNLI: Clinical Scholars National Leadership Institute
CS: Clinical Scholars
CSI: Concept Systems Incorporated
GCM: Group Concept Mapping
MDS: Multi-dimensional scaling
OECD: Organization for Economic Co-operation and Development
RWJF: Robert Wood Johnson Foundation
SDOH: Social Determinants of Health
UNC: The University at North Carolina at Chapel Hill
US: United States
WHO: World Health Organization
WPIP: Wicked Problem Impact Project
