# Supplementary material for: Strengthening healthcare providers’ leadership capabilities, interprofessional collaboration, and systems thinking: a conceptualization of the Clinical Scholars program impact
Source: BMC Med Educ. 2024 Nov 7;24:1277. doi: 10.1186/s12909-024-06240-1 (PMC11542269; doi:10.1186/s12909-024-06240-1)
Supplement: Supplementary file 1 — Supplementary Material 1 [file 12909_2024_6240_MOESM1_ESM.docx]

**Additional file 1. Table A. Brainstorming Statements**

| **Statement number** | **Brainstorming Statement** |
| --- | --- |
| 1 | The Clinical Scholars staff cares about us as people. |
| 2 | The focus on personal development and accountability |
| 3 | The combination of ways to learn and experience leadership skills. |
| 4 | How it emphasizes the dynamics of leadership on ALL levels from the micro level (individual personality assessments) to team dynamics to the macro level (logistics of improving community partnerships). |
| 5 | How the components of the training compliment and reinforce each other-- books, independent online learning modules, in-person learning, executive coaching, webinars, etc. |
| 6 | The diverse input and opinions from peers and instructors that challenge me to think outside the box. |
| 7 | Flexibility in response to a lot of change and trauma experienced as the WPIP was implemented. |
| 8 | The tools and encouragement to be more creative in my work with others. |
| 9 | The challenge of having leaders work in teams with other leaders while navigating the interpersonal dynamics that occur. |
| 10 | Development of a common language around wicked problems that need leadership, vision, implementation, and assessment of results. |
| 11 | The opportunity to think much bigger than one does day to day, by seeing yourself on a larger scale with an ability to actually do something of impact. |
| 12 | The way coaching promoted self-reflection in our TEAM |
| 13 | The way coaching promoted INDIVIDUAL self-reflection |
| 14 | The way coaching addressed burnout both during and after the COVID-19 pandemic |
| 15 | The way personal coaches are role models for how to sustain relationships with the purpose of building healthy communities |
| 16 | Being able to get advice from an experienced leader through executive coaching. |
| 17 | The team coaching increasing our efficacy as a team. |
| 18 | Being able to work in the community setting while being part of the Clinical Scholars program. |
| 19 | The way CS encourages us to make sustainable changes in our communities. |
| 20 | Having access to amazing experiences that we wouldn't have had otherwise. |
| 21 | The sense of community |
| 22 | Inspiring and educational speakers |
| 23 | The CS leadership having foresight for the content that is needed, stating with foundational information, then building layers of content to be used at higher levels of leadership. |
| 24 | Leadership development |
| 25 | Topics that are relevant to our growth as leaders |
| 26 | Multiple opportunities to engage within and across cohorts with activities outside our WPIPs |
| 27 | Exposure to experts in leadership, change management and academia |
| 28 | Exposure to new ideas |
| 29 | Exposure to different ways of thinking |
| 30 | Information that is taught in a new or updated way that helps to update my framework of thinking about my work. |
| 31 | The ability to take theoretical information and apply it to real work situations. |
| 32 | The investment in the teams' projects |
| 33 | The multi-dimensional aspects of leadership and change taught within the program and how those aspects can be applied. |
| 34 | Access to invaluable resources such as knowledgeable speakers, books, funds, intensives, and one another. |
| 35 | Opportunities to learn about myself through the multiple assessments that gave me a comprehensive picture of who I am as a leader. |
| 36 | A new and shared vocabulary to understand and verbalize my own experiences and those of my peers. |
| 37 | The opportunity to practice and demonstrate the skills we are building. |
| 38 | Having vetted speakers and content that make you stretch yourself and explore previously held beliefs. |
| 39 | Providing practical tools and resources |
| 40 | Tools for self-directed learning on our own time/schedule |
| 41 | Training on having tough conversations |
| 42 | The ability to trial ideas and receive group and individual feedback. |
| 43 | The values |
| 44 | Learning about how I can contribute to solving health inequities. |
| 45 | Equity, diversity, and inclusion training |
| 46 | Shared values around diversity, equity, and inclusion. |
| 47 | I now have a network of other equity-minded healthcare leaders both locally and nationally. |
| 48 | A safe and welcoming place to share ideas and learn from others. |
| 49 | A safe and welcoming environment for personal growth and reflection |
| 50 | Being in a space with others who are likeminded. |
| 51 | That it has broadened my perspective. |
| 52 | It has taught me that I can do more than I thought I was capable of. |
| 53 | The sharing from others' expertise and experience broadened my perspective and I was then able to incorporate those learnings into my own practice. |
| 54 | The inspiration that comes from working with outstanding colleagues. |
| 55 | The collective wisdom, knowledge, and experience that is shared by all who participate. |
| 56 | Connections with people doing similar work. |
| 57 | The team approach and cohort model that allows us to build networks with a diverse group of professionals who help us see our challenges from different perspectives. |
| 58 | The interaction with people across different disciplines who are working on the same issues. |
| 59 | Interaction between Fellows from many parts of the country. |
| 60 | Peer support and accountability. |
| 61 | The cohort as a community of engaged learners that encourage one another to grow through challenges. |
| 62 | Opportunities to network with experts in important areas of change leadership |
| 63 | Honest feedback from peers. |
| 64 | Leveraging diversity within our teams. |
| 65 | Facilitating relationships within and outside of our cohort. |
| 66 | Creating interdisciplinary teams. |
| 67 | Breaking down silos between disciplines and sectors. |
| 68 | Interacting with community leaders. |
| 69 | Implementing a WPIP where we can apply what we are learning in real time. |
| 70 | The diversity of the range of the WPIPs. |
| 71 | Flexibility to change our plans/project as needed as we work through our WPIP without loss of funding. |
| 72 | The collaborative team that was built and refined for the WPIP. |
| 73 | The expertise of the CS leadership team. |
| 74 | The Clinical Scholars staff always seems prepared. |
| 75 | The intentionality with which the Clinical Scholars leadership decide on the content for the intensives/retreats. |
| 76 | All the support we receive from the staff who run the program. |
| 77 | The meticulously designed and well-led training at the retreats. The care that the Clinical Scholars team puts into making sure each event is rich and productive is apparent. |
| 78 | The availability of staff and consultants to answer questions and problem solve. |
| 79 | Taking me out of my day to day work to focus on developing leadership skills. |
| 80 | Dedicated time and funds for self-reflection and personal development. |
